# Abnormal Phase Coupling in Parkinson’s Disease and Normalization Effects of Subthreshold Vestibular Stimulation

**DOI:** 10.3389/fnhum.2019.00118

**Published:** 2019-04-03

**Authors:** Soojin Lee, Aiping Liu, Z. Jane Wang, Martin J. McKeown

**Affiliations:** ^1^School of Biomedical Engineering, University of British Columbia, Vancouver, BC, Canada; ^2^Pacific Parkinson’s Research Centre, Vancouver, BC, Canada; ^3^Department of Electronic Science and Technology, University of Science and Technology of China, Hefei, China; ^4^Department of Electrical and Computer Engineering, University of British Columbia, Vancouver, BC, Canada; ^5^Department of Medicine (Neurology), University of British Columbia, Vancouver, BC, Canada

**Keywords:** Parkinson’s disease, electrical vestibular stimulation, EEG, phase locking value, cortical oscillations, sample entropy, sparse discriminant analysis

## Abstract

The human brain is a highly dynamic structure requiring dynamic coordination between different neural systems to perform numerous cognitive and behavioral tasks. Emerging perspectives on basal ganglia (BG) and thalamic functions have highlighted their role in facilitating and mediating information transmission among cortical regions. Thus, changes in BG and thalamic structures can induce aberrant modulation of cortico-cortical interactions. Recent work in deep brain stimulation (DBS) has demonstrated that externally applied electrical current to BG structures can have multiple downstream effects in large-scale brain networks. In this work, we identified EEG-based altered resting-state cortical functional connectivity in Parkinson’s disease (PD) and examined effects of dopaminergic medication and electrical vestibular stimulation (EVS), a non-invasive brain stimulation (NIBS) technique capable of stimulating the BG and thalamus through vestibular pathways. Resting EEG was collected from 16 PD subjects and 18 age-matched, healthy controls (HC) in four conditions: *sham* (no stimulation), EVS1 (4–8 Hz multisine), EVS2 (50–100 Hz multisine) and EVS3 (100–150 Hz multisine). The mean, variability, and entropy were extracted from time-varying phase locking value (PLV), a non-linear measure of pairwise functional connectivity, to probe abnormal cortical couplings in the PD subjects. We found the mean PLV of Cz and C3 electrodes were important for discrimination between PD and HC subjects. In addition, the PD subjects exhibited lower variability and entropy of PLV (mostly in theta and alpha bands) compared to the controls, which were correlated with their clinical characteristics. While levodopa medication was effective in normalizing the mean PLV only, all EVS stimuli normalized the mean, variability and entropy of PLV in the PD subject, with the exact extent and duration of improvement a function of stimulus type. These findings provide evidence demonstrating both low- and high-frequency EVS exert widespread influences on cortico-cortical connectivity, likely via subcortical activation. The improvement observed in PD in a stimulus-dependent manner suggests that EVS with optimized parameters may provide a new non-invasive means for neuromodulation of functional brain networks.

## Introduction

Parkinson’s disease (PD), the second most common neurodegenerative disease (Scandalis et al., 2001), is characterized by motor symptoms such as bradykinesia, tremor, rigidity and impaired balance and gait as well as non-motor complications, resulting primarily from degeneration of dopaminergic neurons in the substantia nigra pars compacta (SNc) (Davie, 2008). Several electrophysiology studies using local field potential (LFP) recordings demonstrated that, in the dopamine-deficient state, the neuronal synchronization in the basal ganglia (BG) is exaggerated at frequencies in the beta range (13–30 Hz) (Brown and Williams, 2005; Eusebio et al., 2009; Litvak et al., 2012; Oswal et al., 2013). These beta oscillations are also highly synchronized with sensorimotor areas (Brown et al., 2001; Marsden et al., 2001; Cassidy et al., 2002; Williams et al., 2002) as well as muscle activity of upper limbs during movement (Marsden et al., 2001). This excessive beta synchronization is considered to be, in part, responsible for the Parkinsonian symptoms and thus reducing the abnormal synchronization with deep brain stimulation (DBS) has shown to be an effective therapy.

Recent fMRI findings have highlighted that large-scale cortical resting-state functional connectivity (rsFC) is altered in PD, possibly as a result of BG impairment effects on cortical-BG networks (Helmich et al., 2010). The striatum, a subcortical region significantly affected with dopamine depletion in PD, has altered FC with inferior parietal, temporal, and motor cortices (Helmich et al., 2010), which supports that PD-induced connectivity changes can be seen beyond local subcortical regions. In addition to effects on BG-cortical FC, impairment in the BG can also alter cortico-cortical connectivity. Diminished interhemispheric connectivity in sensorimotor cortical regions (Seibert et al., 2012) and reduced rsFC in widespread regions including inferior frontal, superior parietal, and occipital regions (Dubbelink et al., 2014) have been shown to be implicated with disease duration and cognitive dysfunctions in PD.

Inferring pathological cortico-cortical connectivity in PD solely based on evidence from fMRI alone may not provide a complete picture, as fMRI has limited temporal resolution. Electrophysiology can provide complementary information as it measures spontaneous synchronous activity of a large population of neurons occurring on a millisecond time scale. A simultaneous LFP-electroencephalography (EEG) study reported that the dynamics of LFP synchrony in STN is related to the dynamics of cortical synchrony (Ahn et al., 2015), and BG DBS modulates cortical phase coupling measured with EEG (Silberstein et al., 2005; Smolders et al., 2013).

One of the most widely-used method to quantify the couplings between oscillatory signals recorded at pairs of electrodes placed on the scalp in EEG is to look at their phase relationships (Klimesch et al., 2008; Fell and Axmacher, 2011). If cortical activities at two different regions are coupled, their phase angle differences tend to be consistent across time. Phase locking value (PLV) quantifies the strength of the phase coupling between two oscillatory signals, bounded between zero and one indicating a completely random and perfectly coupled relationship, respectively. Interregional phase synchronization has been shown to reflect specific neural activity coding different cognitive functions (Klimesch et al., 2001; Hanslmayr et al., 2005), motor behaviors (Andres and Gerloff, 1999; for a review, see Sauseng and Klimesch, 2008) and pathological brain states (Spencer et al., 2004; Sakkalis et al., 2007; Vakorin et al., 2016). However, to date, only a few studies have examined phase-based rsFC across broad cortical regions and different frequency bands in PD (Silberstein et al., 2005; Moazami-Goudarzi et al., 2008; George et al., 2013; He et al., 2017).

Most of the EEG connectivity studies to date have employed magnitude squared coherence. PD subjects exhibit excessive EEG coherence (Silberstein et al., 2005; George et al., 2013), especially in the beta band, in the off-medication condition that is decreased by medication (George et al., 2013). For PD subjects on-medication, enhanced coherence in the frontal regions in the theta (4–6 Hz), beta (12–18 Hz), and gamma (30–45 Hz) (Moazami-Goudarzi et al., 2008) and altered interhemispheric beta coherences in the midtemporal and frontal areas (He et al., 2017) can be observed, indicating the multifarious role of dopamine in the control of oscillatory activity, in and beyond the BG. However, coherence is different from PLV in that it relies on the assumption of linearity and stationarity in the signals and is calculated independently for each frequency, which is then scaled by the amplitudes of the signals. PLV-based connectivity, which do not rely on the strict assumptions underlying coherence, might be more suitable for non-linear and non-stationary dynamics of neural oscillations, and sheds a new light on pathophysiological brain networks as it has not been explored yet in PD.

Recent progress in non-invasive brain stimulation (NIBS) has demonstrated its capability to modulate cortical oscillations (Helfrich et al., 2014; Vossen et al., 2015; Amengual et al., 2017) and interregional couplings, indicating its potential applications as an effective therapeutic technique for PD. Electrical vestibular stimulation (EVS) is a NIBS technique that delivers weak current to the mastoid processes and modulates firing rates of vestibular afferents, which then activates various cortical and subcortical regions including the BG and thalamus (Bense et al., 2001; Utz et al., 2010; Lopez et al., 2012). Similar to transcranial electrical stimulation (tES), EVS stimuli can take the form of direct current (DC), alternating current (AC), or random noise (RN) and stimulation effects vary according to stimulus types. While DC-EVS perturbs perception of orientation and locomotion and has been widely utilized in postural balance control research (St George and Fitzpatrick, 2011), RN-EVS has demonstrated its efficacy in motor functions (Yamamoto et al., 2005; Pan et al., 2008; Lee et al., 2015) and modulation of EEG oscillatory rhythms across broad cortical regions in PD (Kim et al., 2013). It is conceivable, therefore, that EVS may be able to modulate cortical couplings, which has not been explored yet.

To establish the potential of EVS as a therapeutic intervention to modulate abnormal cortical couplings in PD, we investigated whether resting-state cortical couplings, as measured as PLV, were altered in unmedicated PD subjects, and determined if EVS had any normalizing effects. Specifically, we applied three novel EVS stimuli, each restricted to a specific frequency band, to PD and healthy subjects and examined how the different stimuli affected both the strength and temporal variation of aberrant couplings in PD.

## Materials and Methods

### Participants

Twenty PD patients and 22 age- and gender-matched healthy controls (HC) participated in this study. Patients with atypical parkinsonism or other neurological disorders were excluded from the study, and all included PD patients were classified as having mild to moderate stage PD (Hoehn and Yahr Stage 1–2). Four PD and four HC subjects were excluded in the data analysis due to severe muscle artifacts in their EEG recordings. Therefore, 16 PD (7 males; age: 67.3 ± 6.5 years) and 18 HC (9 males; age: 67.6 ± 8.9 years) subjects were included in the analysis (Table 1). All subjects did not have any reported vestibular or auditory disorders and were right-handed. The study protocol was approved by the Clinical Research Ethics Board at the University of British Columbia (UBC) and the recruitment was conducted at the Pacific Parkinson’s Research Centre (PPRC) in UBC. All subjects gave a written informed consent prior to participation.

**Table 1:** Demographic and clinical characteristics of the patients with Parkinson’s disease (PD) and healthy controls (HC).

	PD	HC
Age (years), mean (SD)	67.3 (6.5)	67.6 (8.9)
Gender, n (male/female)	7/9	9/9
Disease duration (years), mean (SD)	7.4 (4.3)	–
^a^UPDRS II, mean (SD)	14.8 (8.1)	–
^b^UPDRS III, mean (SD)	22.1 (8.9)	–
Hoehn and Yahr scale, mean (range)	1.3 (1–2)	–
Levodopa equivalent daily dose (mg), mean (SD) (Tomlinson et al., 2010)	635.9 (356.4)	–


*^*a*^Motor aspects of experiences of daily living. ^*b*^Motor symptoms.*

### Study Protocol

As individuals have inherently subjective perception of EVS, we utilized systematic procedures that have been previously used in determining subliminal current level (Lee et al., 2015). The measured individual threshold level was in the range of 0.23–1.1 mA. After the threshold was determined, the subjects were comfortably seated in front of a computer screen and were instructed to focus their gaze on a continuously displayed fixed target while EEG was being recorded. EEG was first recorded without stimulation for 20 s and EVS were then delivered for a fixed duration of 60 s, followed by an EVS-off period for 20 s (post-stimulation). During the stimulation period, EVS was applied at 90% of the individual threshold level.

EEG was recorded from the subjects in 4 different conditions: *Sham* (no stimulation), EVS1, EVS2 and EVS3 (for details, see section EVS). EEG recording was first performed in the *sham* condition and the EVS conditions were randomly ordered. We allowed a 2 min break between each condition to prevent any potential post-stimulation effects carried over from the previous EVS conditions.

The HC subjects performed the protocol once, whereas PD subjects performed it twice in off-medication (PDMOFF) and on-medication (PDMON) conditions on the same day. The PD subjects stopped taking their normal L-dopa medication at least 12 h, and any dopamine agonists 18 h prior to the EEG recording. United Parkinson’s Disease Rating Scale (UPDRS) Parts II and III were assessed in the off-medication condition. Immediately after finishing the EEG acquisition, they took their regular dose of L-dopa medication and rested for 1 h. After the break, EEG was recorded in the on-medication condition.

### EVS

EVS was delivered through pre-gelled Ag/AgCl electrodes (BIOPAC Systems Inc., CA, United States) placed in bilateral, bipolar fashion over the mastoid process behind each ear. Nuprep^TM^ skin prep gel was used to clean skin for better electrode contact and to reduce resistance during stimulation. Stimulation waveforms were generated on a computer using MATLAB (R2018a, MathWorks, MA, United States) and converted to an analog signal using a NI USB-6221 BNC digital acquisition module (National Instruments, TX, United States). The analog voltage signals were then passed to a constant current stimulator (DS5, Digitimer, United Kingdom), which was connected to the stimulating electrodes.

**FIGURE 1:**
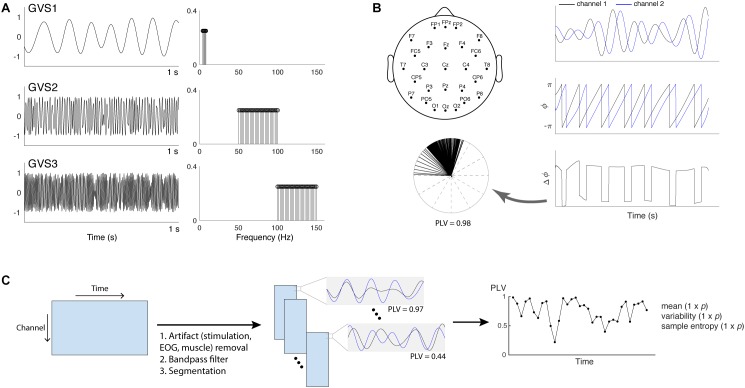
**(A)** Time and frequency plots of the three types of multisine stimulus given at 90% individual threshold level (EVS1: 4–8 Hz; EVS2: 50–100 Hz; EVS3: 100–150 Hz). **(B)** Placement of 27 EEG electrodes and PLV calculation. Hilbert transform is applied to the two signals to extract the instantaneous phases. The phase differences calculated at each time point are represented into unit vectors in the complex plane and PLV is computed to evaluate the spread of the distribution (Lachaux et al., 1999; Mormann et al., 2000). **(C)** The procedure to extract phase locking value (PLV) time series. For each subject, preprocessing steps were first applied to the raw EEG data in order to remove high-voltage stimulation artifacts as well as cardinal artifacts caused by eye movements [electrooculography (EOG)] or muscle movement. The cleaned data were bandpass filtered into four different frequency bands (theta: 4–8 Hz; alpha: 8–13 Hz; beta: 13–30 Hz; gamma: 30–45 Hz) and segmented into epochs. PLV between a pair of electrodes in each epoch was computed to generate the time series, and its mean, variability, and sample entropy were calculated. Each subject has a 1 × *p* vector for the mean, variability and sample entropy (*p* = 1,404 = 351 pairs × 4 frequency bands).

Three multisine signals in different frequency bands (EVS1: 4–8 Hz; EVS2: 50–100 Hz; EVS3: 100–150 Hz) were used (Figure 1A). Multisine signals are designed to concentrate power at a precise number of frequencies within the bandwidth of interest, which is advantageous compared to other excitation signals (e.g., a white noise or swept sine) as there is no spectral leakage. Each multisine signals were designed to have the frequencies of sinusoids (*f_i_*) uniformly distributed every 0.2 Hz and the phases (ϕ*_i_*) chosen to minimize the crest factor using a clipping algorithm (Van der Ouderaa et al., 1988) in order to generate a flat amplitude of the signal and thus improve subject’s comfort:

$$x(t,\;\phi )=a\cdot \sum_{i=1}^{n}\cos\;(2\pi f_{i}t+\phi_{i})$$

where *x*(*t*,ϕ) is the multisine, *a* is the amplitude, *f_i_* and ϕ*_i_* are the frequency and phase, and *i* is the index of each sinusoidal component (e.g., *f_1_, f_2_*, …, *f_n_* = 4.0, 4.2, …, 8.0 Hz for EVS1).

### EEG Recording

Data were recorded from 27 scalp electrodes using a 64-channel EEG cap (Neuroscan, VA, United States) and a Neuroscan SynAmps2 acquisition system (Neuroscan, VA, United States) at a sampling rate of 1 kHz. Recording electrodes were positioned according to the International 10–20 placement standard with one ground and one reference electrode located between Cz and CPz (Figure 1B). Impedances were kept below 15 kΩ using Electro-Gel (Electrode-Cap International, OH, United States). No clipping of EEG was observed during stimulation.

### EEG Preprocessing

The EEG data were bandpass filtered between 3 and 45 Hz using a two-way finite impulse response (FIR) filter (the “eegfilt” function in EEGLAB). High-voltage stimulation artifacts during EVS2 and EVS3 were removed using the digital filters. The artifacts during EVS1 were removed using a quadrature-IVA method (Lee et al., 2019). Data were then re-referenced to the average reference (linked earlobe) and ocular artifacts (EOG) were corrected based on cross-correlation with the reference EOG channels using the AAR toolbox included in EEGLAB. The cleaned EEG data were bandpass filtered into four conventional EEG frequency bands (Groppe et al., 2013): theta (4–8 Hz), alpha (8–13 Hz), beta (13–30 Hz), and gamma (30–45 Hz). The bandpass-filtered data were then segmented into non-overlapping epochs. Epoch sizes were determined such that the epochs include around four cycles at a center frequency of the selected bandwidth (Martin and Schröder, 2016), resulting in epoch sizes of 600, 400, 200, and 100 ms for the theta, alpha, beta, and gamma bands.

### Phase Locking Value (PLV)

PLV evaluates the spread of the distribution of phase angle differences between pairs of electrodes over time (Lachaux et al., 1999; Mormann et al., 2000; Figure 1B). The connectivity is measured from this spread such that strongly clustered phase differences between two electrodes result in the PLV value close to one, indicating a strong connectivity between the signals. If there is no phase dependence, PLV value becomes zero.

To calculate the PLV, instantaneous phase angles were obtained by applying the Hilbert transformation to the bandpass-filtered data. Then, the PLV between two signals A and B was computed as Bruña et al. (2018):

$$PLV_{A,\;B}=\frac{1}{T}\left|\sum_{t=1}^{T}e^{-i(\varphi_{A}(t)-\varphi_{B}(t))}\right|$$

where *T* is the number of time points and φ(*t*) is the instantaneous phase angles of each EEG signal. The PLV was computed for each epoch, resulting in times series of the PLV computed from all pairs of 27 electrodes and the four frequency bands (1,404 time series in total). Three temporal features were extracted from each PLV time series for further analysis: the mean, variability (standard deviation), and sample entropy. Sample entropy is a non-linear measure to quantify the degree of complexity in a time series (Richman and Moorman, 2000), and has been applied to EEG data for clinical application such as classification (Bruce et al., 2009; Kumar et al., 2012) and epilepsy detection (Song et al., 2012). Tolerance (*r*) and window length (*m*) were specified to be 0.3 and 2, respectively, to compute the sample entropy based on Lake et al. (2002) and characteristics of our data sets.

### Sparse Discriminant Analysis

Linear discriminant analysis (LDA) is a classical supervised classification technique that finds the most discriminative projections of a *N* × *p* data in a *p*-dimensional space such that the data projected into the low-dimensional subspace can be well partitioned into *k* classes (Mai, 2013). In biomedical research, it has become an increasingly important topic to perform a classification with a high-dimensional data where the number of variables far exceeds the number of samples. In such high-dimensional settings, LDA cannot be applied directly because of singularity of the sample covariance matrix. To overcome this limitation, various regularized versions of LDA have been proposed (Safo and Long, 2018). Sparse discriminant analysis (SDA) was proposed by Clemmensen et al. (2011) where an elastic net penalty and optimal scoring framework are applied to a high-dimensional data to generate a sparse discriminant vector. The authors demonstrated that SDA outperforms other regularized methods such as shrunken centroids regularized discriminant analysis and sparse partial least squares regression. The details of the algorithm can be found in Clemmensen et al. (2011).

Here, we aim to classify the PDMOFF and HC groups in the baseline resting state (i.e., the *sham* condition) using the PLV features obtained above. The three data sets (mean, variability and sample entropy) have the same high-dimensional settings as each data set has the number of variables (*p* = 1,404) much greater than the number of samples (i.e., subjects). Therefore, we applied SDA to each data set to infer from the sparse discriminant vectors which combination of the electrode pairs and frequency bands are the most important features for the classification of the two groups. As in Clemmensen et al. (2011), we created the training set consisted of 26 subjects (12 PDMOFF and 14 HC) and the test set of eight subjects (4 PDMOFF and 4 HC subjects) and the tuning parameters for SDA (i.e., λ and γ for regularization penalties) were chosen using leave-one-out cross-validation (LOOCV) on the training data. The models with the selected parameters were evaluated on the test data.

In the subsequent analyses, we investigated effects of L-dopa medication on the PLV features by applying the sparse discriminant vectors obtained from the above SDA to the data sets of the PDMON group in the *sham* condition. In the same manner, effects of EVS on the PLV features were evaluated by applying the same sparse discriminant vectors to the data sets in the EVS conditions.

### Statistical Analysis

A one-way ANOVA was performed to compare the PLV features between groups followed by *post hoc* Tukey’s honestly significant difference (HSD) test for multiple comparison correction. To evaluate effects of EVS on the PLV features within a group, a repeated measures (rm) ANOVA, with stimulation condition (*sham*, EVS1, EVS2, and EVS3) as the within-subject factor, was performed followed by *post hoc* Tukey’s HSD test for multiple comparison correction. The rm ANOVA analysis was performed for online and after-effect conditions, respectively.

## Results

### SDA Classification Results and Selected Features

**FIGURE 2:**
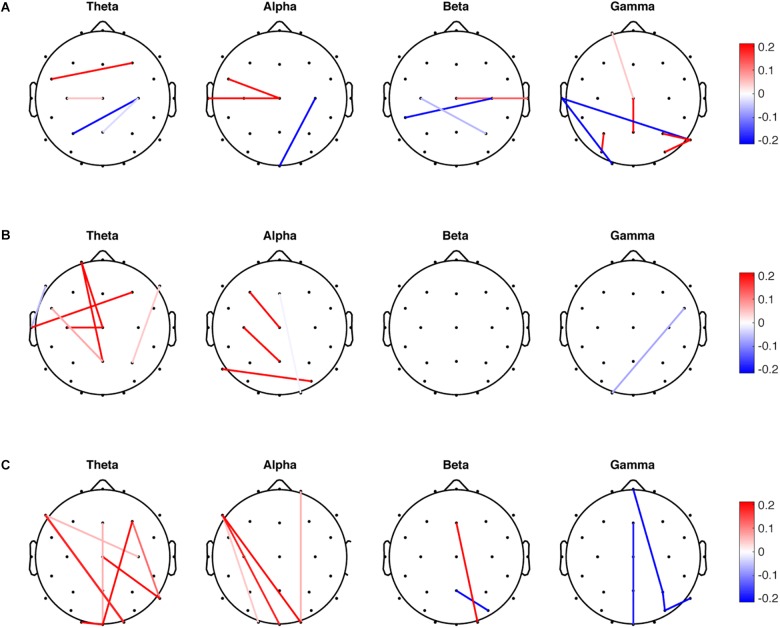
Non-zero features selected by sparse discriminant analysis (SDA). SDA was applied to the mean, variability and entropy data sets, respectively, to discriminate the PDMOFF and HC groups. The non-zero weights in the sparse discriminant vectors are presented in the scalp maps. **(A)** Weights for the 17 selected features from the mean PLV data set. **(B)** Weights for the 12 selected features from the PLV variability data set. **(C)** Weights for the 17 selected features from the PLV entropy data set.

**FIGURE 3:**
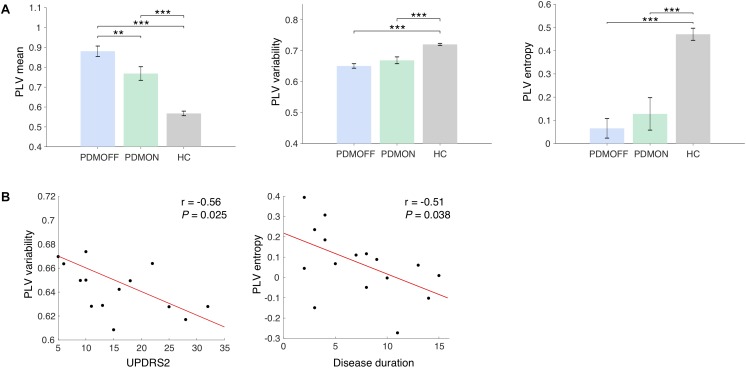
**(A)** Group comparison of the discriminant component obtained from the SDA. The discriminant components were obtained by multiplying the discriminant vectors to the data sets from the *sham* condition. Bars and error bars indicate group means and s.e. Significant *P*-values from one-way ANOVA with *post hoc* Tukey’s HSD test are indicated (^∗∗^*P* < 0.01; ^∗∗∗^*P* < 0.001). **(B)** Pearson correlations with clinical scores. The PLV variability and entropy of the PDMOFF subjects are significantly correlated with UPDRS2 and disease duration, respectively.

SDA was performed for the mean, variability, and entropy PLV data sets independently to discriminate the PDMOFF and HC groups. Since there are two classes in the data, only one discriminant vector was obtained from each SDA. For the mean PLV data set, LOOCV on the training data resulted in the selection of 17 non-zero features (1.2%) out of total 1,404 features (Figure 2A). There were both negative and positive weights for the selected features in each frequency band. Since the transformed PLV mean was greater for the PDMOFF (Figure 3A) than the HC group, the positive weights were interpreted as cortical couplings exaggerated in the PDMOFF group. 35% of the selected features were associated with Cz over a broad frequency bandwidth, and the PDMOFF group had a stronger coupling strength for the features. In contrast, the features related to C4 had negative weights, indicating that these couplings are attenuated in the PDMOFF group. In the gamma band, decreased long-distance connectivity in the left temporal region (T7-O1 and T7-P8) and increased short-distance connectivity in the parietal region (P3-PO5, P8-P4, and P8-PO6) were found to be related to the PDMOFF group. The training and test classification accuracy (fraction of correctly classified) were both 100%.

For the PLV variability data set, 12 non-zero features (0.85%) were selected and the largest number of the selected features was found in the theta band (Figure 2B), followed by the alpha and gamma bands. Note that positive weights are associated with the lower connectivity variability of the PDMOFF group because the transformed variability is lower for the PDMOFF group (Figure 3A). Decreased variability in the PDMOFF group was mostly associated with the frontal electrodes in the theta band and with F3-Cz, C3-Pz, and P7-PO6 in the alpha band. The classification accuracy for the training and test data sets were 100 and 87.5%, respectively.

The SDA on the PLV entropy data set selected 17 non-zero features (1.2%) and most of them were long-distance connectivity. Note that positive weights are associated with the connectivity with lower entropy for the PDMOFF group. In the theta and alpha bands, the entropy of the selected features was lower whereas in the gamma band the entropy was higher for the PDMOFF group compared to the HC group. In the beta band, the PDMOFF group had a lower entropy for Fz-O2 and higher entropy for Pz-PO6 than the HC group. The training and test classification accuracy were 96 and 87.5%, respectively.

### Group Comparison of Baseline PLV Features

The SDA discriminant vectors were applied to the data sets obtained from the PDMON group, and the group means of the transformed data are compared in Figure 3A. Significant group differences were found for the PLV features [PLV mean: *F*(2, 47) = 41.68, *P* < 0.001; PLV variability: *F*(2, 47) = 23.46, *P* < 0.001; PLV entropy: *F*(2, 47) = 60.59, *P* < 0.001]. The PLV mean for the PDMOFF group was significantly higher than the HC group (*P* < 0.001), which was decreased by L-dopa medication (*P* < 0.01). The PLV variability was significantly lower in the PDMOFF compared to the HC group (*P* < 0.001), and the lower variability was associated with higher UPDRS2 scores (i.e., more severe difficulties of daily motor activities) (*r* = -0.56, *P* = 0.025; Figure 3B). The medication slightly improved the variability in the PD subjects but the changes did not reach statistical significance (*P* = 0.096). The entropy of the PDMOFF group was lower than the HC group (*P* < 0.001) and the lower entropy was related to a longer disease duration (*r* = -0.51, *P* = 0.038; Figure 3B). The medication did not improve the PLV entropy (*P* = 0.41).

### Online- and After-Effects of EVS

**FIGURE 4:**
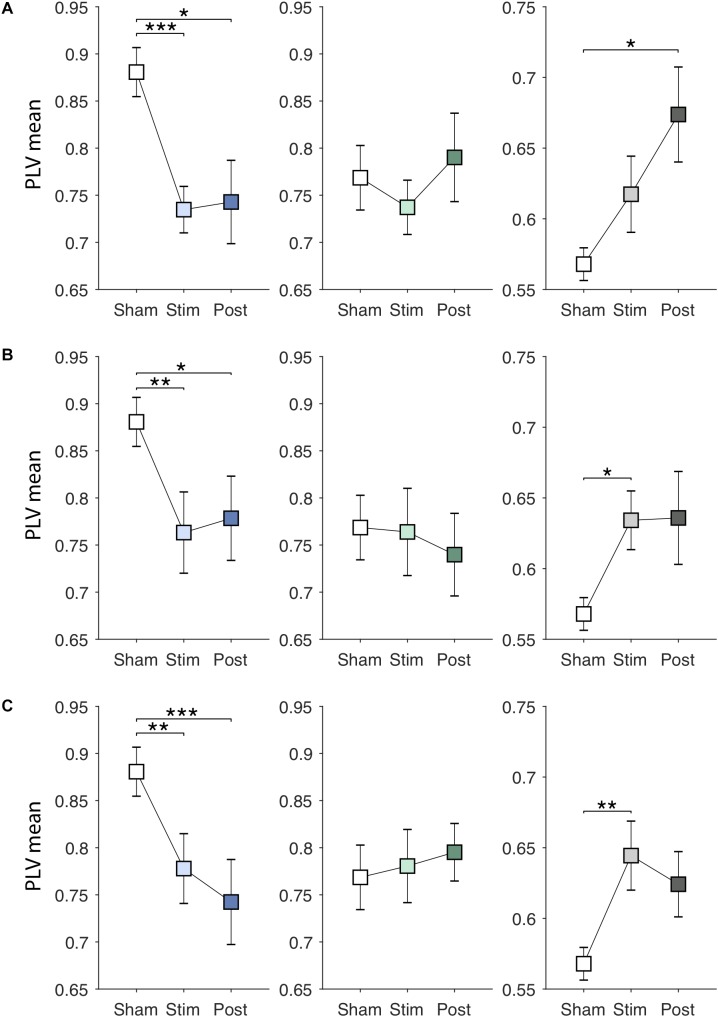
Effects of EVS on the PLV mean. The PLV mean values in the *sham* condition are identical to those in Figure 3A. The PLV mean values in the stimulation (60 s) and post-stimulation period (20 s) were obtained in the same manner by multiplying the discriminant vector to the corresponding data sets. In each row, from the left, the results for the PDMOFF (blue), PDMON (green), and HC (gray) groups are presented in each panel. Significant *P*-values from repeated measures ANOVA with *post hoc* Tukey’s HSD test are indicated (^∗^*P* < 0.05; ^∗∗^*P* < 0.01; ^∗∗∗^*P* < 0.001). **(A)** EVS1 effects. **(B)** EVS2 effects. **(C)** EVS3 effects.

Next, EVS effects on the PLV features were investigated. Specifically, we examined whether the effects are dependent on the stimulus types and sustained even after the stimulation ceases. Figure 4A–C show changes in the PLV mean for each group induced by EVS1, EVS2, and EVS3, respectively. The PLV mean was significantly modulated during stimulation in PDMOFF [*F*(3, 45) = 11.16, *P* < 0.001] and HC [*F*(3, 51) = 3.81, *P* < 0.05] groups. All stimuli decreased the PLV mean in the PDMOFF group compared to the *sham* condition (EVS1: *P* < 0.001; EVS2: *P* < 0.01; EVS3: *P* < 0.01), making it closer to the HC group, and the effects lasted in the post-stimulation period. EVS1 decreased the mean PLV greater than the other two stimuli and there was no continuing decrease in the post-stimulation period whereas EVS3 decreased the mean PLV less than EVS1 during stimulation and the effect continued in the post-stimulation period. In contrast, we found the opposite EVS effects for the HC group where EVS increased the PLV mean (EVS2: *P* < 0.05; EVS3: *P* < 0.01). No significant effects of EVS were found in the PDMON group.

**FIGURE 5:**
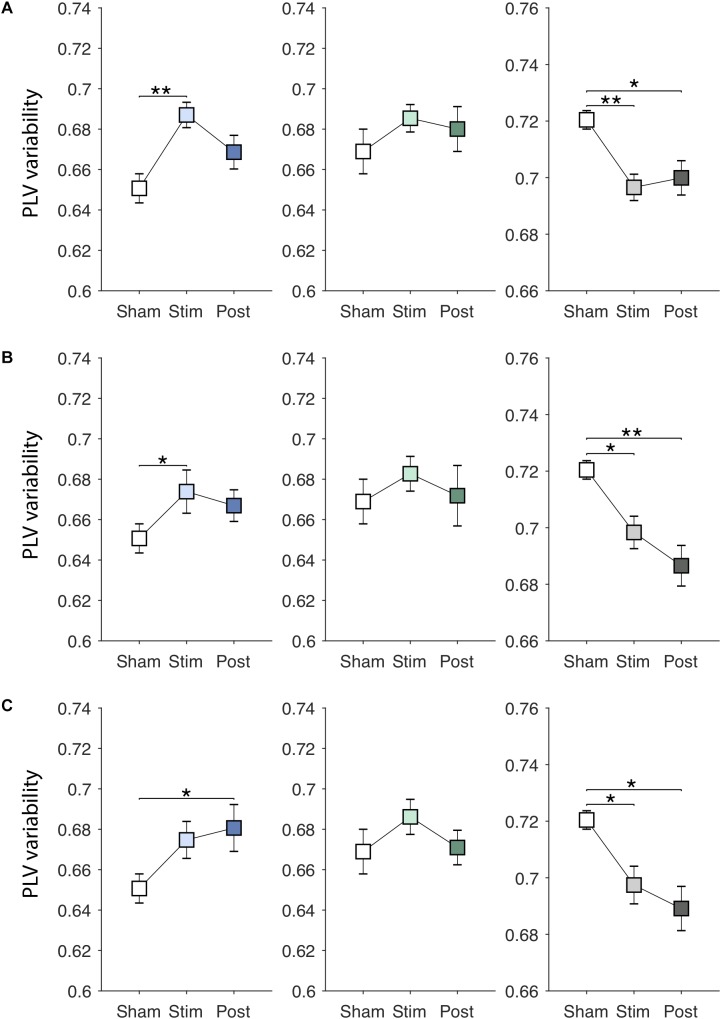
Effects of EVS on the PLV variability. The PLV variability values in the *sham* condition are identical to those in Figure 3A. Descriptions for the arrangement of the plots and statistical significance are same as in the Figure 4. **(A)** EVS1 effects. **(B)** EVS2 effects. **(C)** EVS3 effects.

EVS effects on the PLV variability are presented in Figure 5A–C. There were significant online effects of stimulation on the PLV variability in PDMOFF [*F*(3, 45) = 4.43, *P* < 0.01] and HC [*F*(3, 51) = 4.62, *P* < 0.01] groups. EVS1 and EVS2 were found to have positive effects on the PDMOFF group, increasing the variability during stimulation (EVS1: *P* < 0.01; EVS2: *P* < 0.05). Similar to the effects on the PLV mean, EVS1 induced the greatest increase in the variability during stimulation and the increased value tends to return to the baseline after the stimulation ceased whereas the effects of EVS2 and 3 were less during stimulation but lasted longer than that of EVS1. In the HC group, we found decreases in the PLV variability induced by EVS (EVS1: *P* < 0.01; EVS2: *P* < 0.05; EVS3: *P* < 0.05). EVS1 decreased the variability during the stimulation and the effect lasted in the post-stimulation period. EVS2 and EVS3 appeared to further decrease the variability in the post-stimulation period. For the PDMON group, all stimuli increased the PLV variability but the effects did not reach the statistical significance.

**FIGURE 6:**
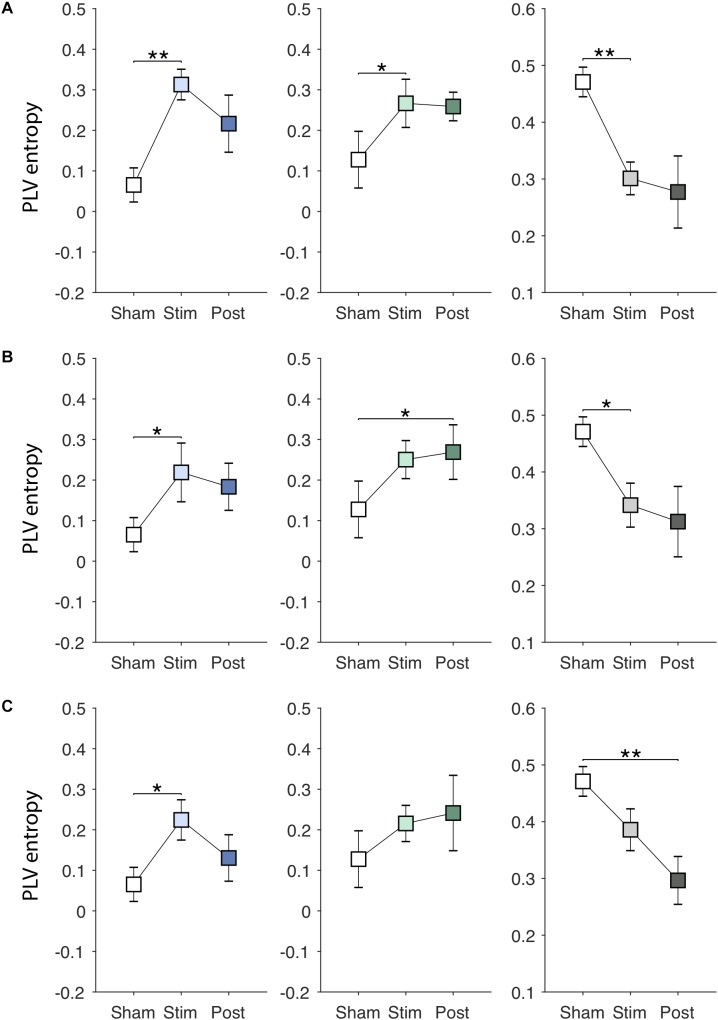
Effects of EVS on the PLV entropy. The PLV entropy values in the *sham* condition are identical to those in Figure 3A. Descriptions for the arrangement of the plots and statistical significance are same as in the Figure 4. **(A)** EVS1 effects. **(B)** EVS2 effects. **(C)** EVS3 effects.

Figure 6A–C show EVS effects on the PLV entropy. The PLV entropy was significantly modulated during stimulation in PDMOFF [*F*(3, 45) = 4.65, *P* < 0.01], PDMON [*F*(3, 45) = 3.12, *P* < 0.05], and HC [*F*(3, 51) = 4.25, *P* < 0.01] groups. We found that all stimuli increased the entropy significantly in the PDMOFF group (EVS1: *P* < 0.01; EVS2: *P* < 0.05; EVS3: *P* < 0.05), bring it closer to the HC group. The effects were greatest during stimulation and diminished in the post-stimulation period, and EVS1 increased the largest amount of the entropy. For the PDMON group, EVS1 (*P* < 0.05) and EVS2 (*P* < 0.05) increased the entropy significantly. While not statistically significant, increases in the entropy were also found during and post-EVS3 compared to the *sham* condition. The PLV entropy of the HC group changed in the opposite direction by EVS compared to the PD groups. Significant decreases in the entropy was observed with all stimuli [EVS1 (*P* < 0.01), EVS2 (*P* < 0.05) and EVS3 (*P* < 0.01)].

## Discussion

We investigated phase-based cortical connectivity in resting EEG in PD. To our knowledge, this is the first study that examined connectivity dynamics in PD by characterizing temporally fluctuating cortico-cortical couplings over broad frequency bands. The results from the current study on the time-varying connectivity provide novel insights into altered cortical dynamics derived from pathological BG changes in PD.

### Disrupted Cortical Coupling Strength in the Motor Regions

We found most changes in cortical coupling strength associated with PD (Figure 2A; 11 out of 17) were in key motor and parietal regions, including over the primary motor cortex (M1), supplementary motor area (SMA), premotor area (PMA), and superior parietal regions, which was in line with previous findings (Otten et al., 2012). Typically, a common finding of pathological synchronization in PD is hypersynchronization of the cortical regions in the beta range (Silberstein et al., 2005; George et al., 2013; Pollok et al., 2013). This appears to be related to exaggerated beta synchronization within the BG and between the BG and motor cortical regions (Brown, 2007; Jenkinson and Brown, 2011; Brittain and Brown, 2014). However, growing evidence indicates that PD has more complex influences on motor networks beyond excessive beta synchronization (Wu et al., 2009, 2011). There is altered cortical oscillatory activity in other bands beside beta (Bosboom et al., 2006; Stoffers et al., 2007). On the other hand, there is substantial agreement that therapeutic DBS (Pollok et al., 2013) and dopaminergic medication (Wu et al., 2009; Heinrichs-Graham et al., 2014; Tahmasian et al., 2015) have normalizing effects on rsFC of motor networks in PD. Consistent with these findings, our results demonstrated that the altered connectivity found in the PDMOFF group was normalized by both medication and EVS to a similar extent.

### Variability and Entropy of PLV in the Theta Band

The altered variability and entropy of PLV in the PD group were mostly found in the theta band (Figure 2B,C), which may reflect abnormalities in thalamocortical dynamics. The ventral anterior (VA) and anterior part of ventral lateral (VLa) thalamic nuclei are the major recipients from the globus pallidus internus (GPi) via pallidothalamic tracts that are crucially involved in motor disorders such as PD (Gallay et al., 2008). Simultaneously-recorded LFP in the VA and VLa nuclei and EEG on the scalp from PD subjects demonstrated the highest coherence in the theta band (4–9 Hz), in particular in the frontal region of both hemispheres (Sarnthein and Jeanmonod, 2007). Thalamocortical interaction may thus be a major influence in generation of frontal theta activity in PD, and possibly also healthy controls, but we typically do not have LFP recordings from healthy subjects. Multimodal functional imaging studies in healthy human and animal models suggest that the thalamus is critically involved in generating and modulating activities in the cortex (Klimesch, 1999; Schreckenberger et al., 2004; Hughes and Crunelli, 2005; Klimesch et al., 2007). The enhanced synchronization in the theta band of the thalamus and frontal cortical region may be reflective of pathological changes in PD. Together, we conjecture that the increased mean and reduced variability in theta that we observed in PD subjects was a consequence of excessive synchronization between thalamocortical structures.

### Variability and Entropy of PLV in the Alpha Band

The dominant frequency in the human EEG under rest is in the alpha frequency band (8–13 Hz). Alpha oscillations are known to be affected by visual and auditory stimuli (Hari et al., 1997) and change during voluntary movement (Pfurtscheller et al., 2006). A large body of evidence has also demonstrated the critical role of alpha rhythms in attention as well as various cognitive functions (Klimesch, 1999). The dynamic change of alpha activity reflects a variability of states with enhanced and reduced cortical excitability, facilitating the brain’s responses to surrounding stimuli (Garrett et al., 2013). Several studies have shown that brain signal variability/complexity can serve as an important discriminator for clinical comparisons. For example, EEG entropy is related to brain maturity, as adults have higher entropy compared to children and adolescents (Lippe et al., 2009). Higher entropy is also correlated with better performance on a working memory task (McIntosh et al., 2008). Schlee et al. (2014) found reduced variability of alpha activity during rest over the temporal cortex for subjects with tinnitus compared to controls. Similarly, the reduced variability and complexity of the cortical couplings of the PD groups we observed may be related to diminished motor and cognitive adaptability, as executive cognitive functions such as set shifting, divided or alternating attention and dual tasking (e.g., combining walking with another task) are impaired in PD (Muslimovic et al., 2007; Aarsland et al., 2010; Watson and Leverenz, 2010). Although the mechanisms responsible for these symptoms have not been fully accounted for, dopaminergic depletion in the striatum disrupts the parallel organization of cortico-striatal circuits, resulting in more widespread instead of domain-specific involvement of striatal activity and loss of the normally segregated circuits (Bergman et al., 1998; Calabresi et al., 2015; Nieuwhof et al., 2017). Our results together with the close relationships between cortico-striatal circuits and cortical alpha oscillations (Laufs et al., 2003; Slagter et al., 2017) warrant future studies to further elucidate the functional implications of the impaired alpha dynamic couplings we have demonstrated here.

### PLV Sample Entropy Is Higher in the Long-Range Gamma Activity in PD

We found that the connectivity in the gamma band was more irregular in the PD group than the HC group (Figure 2C). The binding of cortical regions together via synchronization of gamma oscillations between neuronal populations, is implicated in numerous cognitive processes (Fries, 2009; Sohal, 2012). In voluntary movement, for example, synchronization of cortical gamma oscillations prior to movement onset has been described as representing active information processing (Pfurtscheller et al., 1993; Salenius et al., 1996) and considered to serve as a prokinetic signal (Brittain and Brown, 2014). Abnormal gamma oscillations in the motor cortex in PD have been reported (Litvak et al., 2012; Nowak et al., 2018). However, resting-state gamma oscillations and connectivity in PD remain largely unknown. The mechanism underlying generation of the gamma oscillations are known to be critically involved with excitatory post-synaptic potentials (EPSPs) of gamma-aminobutyric acid (GABA)ergic interneurons and their intact function of fast-spiking (Fuchs et al., 2001; Vreugdenhil et al., 2003; Hajos et al., 2004). Thus, alterations in function of GABAergic interneurons could be inferenced from gamma-band oscillations at the macroscopic level. The fast-spiking interneurons are modulated by neurotransmitters including acetylcholine (Fries, 2009; Teles-Grilo Ruivo and Mellor, 2013; Tremblay et al., 2016) and serotonin (Fries, 2009; Puig et al., 2010), and there is robust evidence demonstrating deficits in the cholinergic and serotoninergic systems in PD contributing to various aspects of parkinsonian pathophysiology including motor symptoms, gait dysfunction, cognitive decline, autonomic dysfunction (for review, see Perez-Lloret and Barrantes, 2016). Therefore, it is likely that the disrupted neurotransmitter systems in PD cause alterations in the activities of fast-spiking interneurons, subsequently resulting in pathological cortical couplings in the gamma band in PD.

### Normalizing Effects of EVS and Potential Mechanisms

In this study, we demonstrated that EVS normalizes the mean, variability and entropy of PLV in PD subjects during stimulation and the extent and duration of the effects were dependent on the stimulation frequencies (Figure 4–6). Modulatory effects of EVS on the cortical oscillatory activity were reported in prior EEG studies that noisy stimulus (pink noise in 0.1–10 Hz) decreased gamma oscillatory activity in the lateral regions and increased the beta and gamma activity in the frontal region (Kim et al., 2013), and altered interhemispheric coherence (Lee et al., 2017). To our knowledge, effects of high-frequency multisine EVS (>50 Hz) on cortical activity have not been explored yet in humans and the results presented in this study provide valuable information on how the effects would differ from low-frequency EVS that has been used in prior behavior and neuroimaging studies. We found two characteristics of effects induced by EVS2 and EVS3 on PLV. First, their effects were similar to EVS1 in the sense that the direction of changes (i.e., increase or decrease in the PLV features) was the same. We did not find a frequency specific increase or decrease in the PLV value in both the PD and HC groups. Second, the extent of changes was less compared to EVS1 during the high-frequency stimulation but lasted longer in the post-stimulation period. This was observed in the PDMOFF group for all the PLV measures and in the HC group for the variability and entropy. For the PDMON group, the EVS effects were less significant, indicating the processing of vestibular inputs in the thalamus and BG (Lopez and Blanke, 2011; Stiles and Smith, 2015; Wijesinghe et al., 2015) is dependent on the dopaminergic level of the BG.

Modulation of firing rates of vestibular afferents by externally applied electrical current will alter directly the vestibular nuclei activities in the brain stem, and eventually multiple cortical areas through the thalamocortical vestibular system. Thus, understanding vestibular information processing regarding varying frequency contents at the vestibular nuclei and thalamus is critical to comprehend above findings. A prior study that examined spiking rates of the guinea pig medial vestibular nuclei (MVN) reported that two types of neurons having different characteristics of afterpotentials responded to current inputs differentially according to the frequency content (1–30 Hz) (Ris et al., 2001). It was shown that spontaneous firing rates of type A neurons was well modulated by only low-frequency (<10 Hz) current inputs and the spiking rates become irrelevant to the current input at high frequencies whereas type B neurons tended to fire in synchrony better when the stimulation frequency was higher, which demonstrates existence of signal transformation at the vestibular nuclei level to a certain extent in that type A neurons act like a low-pass filter (du Lac and Lisberger, 1995; Ris et al., 2001) whereas type B neurons act as signal detectors with greater sensitivity to external stimuli at high frequencies.

Considering functional roles of the thalamic nuclei playing integrative and modulatory roles in sensorimotor processing (Tyll et al., 2011), it is likely that further transformation of the modified signal transmitted from the vestibular nuclei occurs in the thalamus. The VA, VL, ventral posterior lateral (VPL), ventral posterior medial (VPM), intraminar nuclei and geniculate bodies of the thalamus receive primary afferents from the vestibular nuclei and play a critical role in processing vestibular information (Bucher et al., 1998; Bense et al., 2001; Stephan et al., 2005; Meng et al., 2007; Wijesinghe et al., 2015). These thalamic nuclei also receive a range of different afferents from peripheral sensory, subcortical, and cortical regions, and process the different types of information before sending the refined signals to the cortex. This may also explain the interaction between EVS and L-dopa medication as observed in the PDMOFF and PDMON groups as the thalamic nuclei processing vestibular information would be receiving differential inputs from the BG according to dopamine levels. Together, unlike the transcranial electrical or magnetic stimulation that directly target cortical regions of interest, influences of EVS on cortical activities are much more indirect. Our results suggest that although the frequency contents of current input to the peripheral vestibular nerve vary considerably, alterations of the refined higher-level multisensory information transmitted from the thalamic nuclei to the broad cortical regions may be relatively consistent.

### Limitations

In the current work, the post-stimulation effects were only evaluated for the first 20 s after stimulation ceased and there may be potential confounding effects if the after effects persist much longer. Aftereffects of EVS on cortical activation have not been fully investigated yet. Delayed responses in the beta and gamma power in frontal regions was reported to appear 20–25 s after 72-s EVS, but lasted only for several seconds. Based on prior studies reporting aftereffects of invasive (Wingeier et al., 2006) and non-invasive stimulation (Strüber et al., 2015) and the short duration of weak current EVS used here, we concluded that the break time and randomly-ordered trials were sufficient to avoid confounding effects.

## Conclusion

In conclusion, in this resting-state EEG study, we demonstrated that connectivity strengths in the sensorimotor region, and variability and complexity of the time-varying cortico-cortical connectivity are affected in PD, and improved by subthreshold EVS. Furthermore, the magnitude and duration of the improvement was found to vary depending on the stimulation frequency and the subjects’ dopamine level. The findings from the current study provide valuable information that thalamic functions of integrating subcortical afferent inputs and thalamocortical projections to the cortex play a critical role in the mechanism of the EVS effects, and warrant further investigation of EVS as a potential therapy in PD.

## Data Availability

The raw data supporting the conclusions of this manuscript will be made available by the authors, without undue reservation, to any qualified researcher.

## Author Contributions

SL and MM designed the study. SL conducted the study, performed the data analysis, and wrote the first draft of the manuscript. AL and ZW contributed to interpretation of data for the work. All authors contributed to manuscript revision, read, and approved the submitted version.

## Conflict of Interest Statement

The authors declare that the research was conducted in the absence of any commercial or financial relationships that could be construed as a potential conflict of interest.
